# Supplementary material for: Chromothripsis during telomere crisis is independent of NHEJ, and consistent with a replicative origin
Source: Genome Res. 2019 May;29(5):737–49. doi: 10.1101/gr.240705.118 (PMC6499312; doi:10.1101/gr.240705.118)
Supplement: Supplemental Material [file supp_gr.240705.118_Supplemental_file_1.zip › contigs/annotated_contigs/DB47/contig.2.DB47_length_320_mean_cov_14.78125.docx]

**DB47_length_320_mean_cov_14.78125**

```
CGCACAAACACACTGCCCCCATAACACTGTCCTCAAACCCCG|TTTGACAGACAAACATCCAGAACC|CAGCAGCAGTGTTCTGGAATC
                                           >chr2:132835124-132835195 - E=3e-13 p=0e+00
CTATGTGAGGTACAAACATTCAGAAC|ACTGCAGAAGTGTTCTGGAATCTCCTGTGAGGAAGAAACTTTTAGACCTTCATAGAAGTGGT
>chr17_gl000205_random:169063-169317 - E=9e-133
CTGGATTCCGATGTGATGGACAAACATTTAGACCCCAGCAGCAGTGTTCTGGAATCCTATGTGAGAGACAAACATTCAGAACCTCGTAG

CAGTGTTCTGGAATCCTATGTGAGGGACAAACCCTCAGAACCCAGCAACTGTATTC
```
